# Specialized Cilia in Mammalian Sensory Systems

**DOI:** 10.3390/cells4030500

**Published:** 2015-09-11

**Authors:** Nathalie Falk, Marlene Lösl, Nadja Schröder, Andreas Gießl

**Affiliations:** Department of Biology, Animal Physiology, University of Erlangen-Nuremberg, 91058 Erlangen, Germany; E-Mails: Nathalie.Falk@FAU.de (N.F.); Marlene.Loesl@FAU.de (M.L.); Nadja.Schroeder@fau.de (N.S.)

**Keywords:** primary cilia, intraflagellar transport, kinocilium, inner ear, olfactory epithelium, retina, connecting cilium

## Abstract

Cilia and flagella are highly conserved and important microtubule-based organelles that project from the surface of eukaryotic cells and act as antennae to sense extracellular signals. Moreover, cilia have emerged as key players in numerous physiological, developmental, and sensory processes such as hearing, olfaction, and photoreception. Genetic defects in ciliary proteins responsible for cilia formation, maintenance, or function underlie a wide array of human diseases like deafness, anosmia, and retinal degeneration in sensory systems. Impairment of more than one sensory organ results in numerous syndromic ciliary disorders like the autosomal recessive genetic diseases Bardet-Biedl and Usher syndrome. Here we describe the structure and distinct functional roles of cilia in sensory organs like the inner ear, the olfactory epithelium, and the retina of the mouse. The spectrum of ciliary function in fundamental cellular processes highlights the importance of elucidating ciliopathy-related proteins in order to find novel potential therapies.

## 1. Introduction

**Figure 1:**
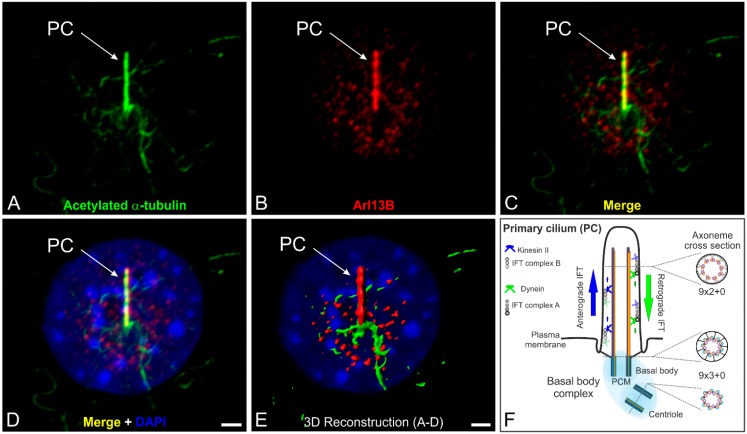
The primary cilium and its intraflagellar transport machinery. (**A**–**D**) Triple labeling of acetylated α-tubulin (**A green**, marker for the ciliary axoneme), ADP-ribosylation factor-like 13B (Arl13B; **B red**, marker for the ciliary membrane) and DAPI (**D blue**) as a nuclear marker in serum-starved NIH 3T3 mouse fibroblasts; (**C**,**D**) Merged images showing the localization of acetylated α-tubulin and Arl13B at a single primary cilium projecting from the cell surface; (**E**) 3D reconstruction of the non-motile primary cilium demonstrating that Arl13B (**red**) ensheathes the axoneme of the primary cilium (PC) labeled with an antibody against acetylated α-tubulin (**green**); (**F**) Schematic of the primary cilium and its intraflagellar transport machinery. The primary cilium is divided into the basal body complex (shaded in **blue**) and the membrane-bound axoneme (9 × 2 + 0 microtubule configuration) extending from the surface. The basal body complex comprises the basal body and its centriole (9 × 3 + 0 structure) enclosed by the pericentriolar material (PCM). Elongation of the axoneme is mediated by the intraflagellar transport (IFT) machinery. Anterograde IFT from the base to the tip of the cilium depends on IFT B proteins and the microtubule plus-end-directed kinesin II motor protein family. Retrograde IFT from the tip to the base of the cilium depends on IFT A proteins and the minus-end-directed cytoplasmic dynein 2 motor protein. Scale bars: 5 µm (**A**–**D**), 2 µm (**E**).

Cilia and flagella are highly conserved microtubule-based organelles consisting of nine peripheral doublet microtubules (9 × 2) that project from the surface of eukaryotic cells. Doublet microtubules are specialized structures composed of one complete microtubule (the A tubule) linked to an incomplete second microtubule (the B tubule) with fewer protofilaments [1]. There are four types of cilia: motile cilia with a central pair of microtubules (9 × 2 + 2 organization; e.g., respiratory cilia, ependymal cilia), motile cilia without a central pair of microtubules (9 × 2 + 0 organization; e.g., nodal cilia), non-motile cilia with a central pair of microtubules (9 × 2 + 2 organization; e.g., kinocilium of hair cells), and non-motile cilia normally lacking the central pair of microtubules (9 × 2 + 0 organization; e.g., photoreceptor-connecting cilium) [2,3,4]. Additionally, numerous vertebrate cell types possess one single primary cilium, a non-motile cilium, located on their surface (Figure 1A–E).

In most non-dividing cells the centrioles of the centrosome migrate to the cell surface, where the mother centriole forms a basal body which anchors the nine peripheral doublet microtubules and organizes formation of the axoneme (Figure 1F). Elongation of the membrane-bound axoneme is mediated by the intraflagellar transport (IFT) machinery, which transports axoneme precursors to the distal tip for assembly. Anterograde trafficking from the base to the tip of the cilium (minus to plus end) depends on microtubule plus-end-directed kinesin II motor proteins associated with IFT B protein complexes. However, the minus-end-directed cytoplasmic dynein 2 motor protein together with IFT A proteins appear to be required for retrograde trafficking (plus to minus end). IFT complex A and B comprise 17 highly conserved proteins [5,6] (Figure 1F). The IFT system has enabled eukaryotic cells to move proteins to the tip of the cilium and back in a specific and well-regulated manner. The evolutionarily conserved mechanism of IFT, which was first described in the laboratory of Joel Rosenbaum in the green alga *Chlamydomonas reinhardtii* [7], is essential for assembly and maintenance (exclusively shown in *Chlamydomonas* so far) of these structures in all species [8,9,10]. IFT is a highly investigated and exceedingly complex process. For more details see e.g., [11,12].

In the last 15 years the cilium has emerged as a key organelle in numerous physiological and developmental processes. Cilia generate flow of mucus and cerebrospinal fluid, as well as leftward flow in the vertebrate node (motile cilia) [13,14,15,16]. The cilium also acts as a sensor for extracellular signals (non-motile primary cilia) and plays a part in several important pathways, e.g., Hedgehog, Wnt (wingless-Int-1), and PCP (planar cell polarity) signaling [1]. Genetic defects in cilia formation, maintenance, or function underlie a wide array of human diseases including retinitis pigmentosa, polycystic kidney disease (PKD), polydactyly, primary ciliary dyskinesia (PCD), and developmental delays, all of which are collectively called ciliopathies [17] (Table 1). It is increasingly important to elucidate the function of ciliary proteins associated with these ciliary defects in order to find potential therapies.

An organism’s perception of its natural environment is dependent on sensory function. As sensors of our environment, cilia are involved in fundamental biological processes such as hearing, olfaction, and photoreception. The corresponding organs can therefore be affected by mutations in ciliary proteins. These ciliopathies are caused by deficient formation and dysfunction of cilia leading to sensory impairments. Mutations in genes encoding ciliary proteins mostly result in disrupted cilia formation up to degeneration of the entire ciliated cell [18,19,20,21]. Patients with ciliopathies affecting the inner ear are often deaf and/or exhibit balance difficulties [22]. Patients with an affected olfactory epithelium often show a complete loss of smell (anosmia) [23,24], and patients with retinal ciliopathies often become blind (retinal degeneration) [25,26,27]. Impairment of more than one sensory organ results, for instance, in Bardet-Biedl [28,29] or Usher syndrome [30,31], two autosomal recessive genetic diseases (Table 1).

## 2. Cilia in the Inner Ear of Mammals

**Figure 2:**
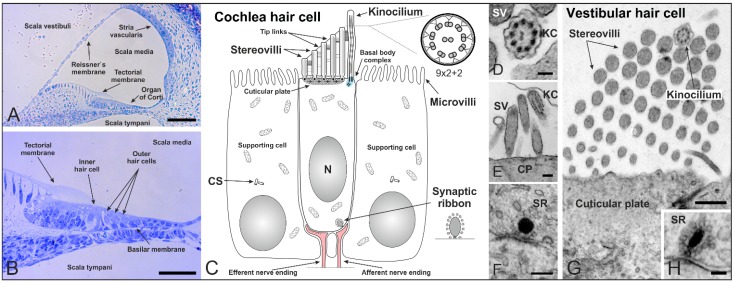
Cilia in the inner ear of mammals shortly after birth. (**A**,**B**) Cross section of a mouse cochlea at P5 (postnatal day 5) stained with toluidine blue. The cochlea is divided into three fluid-filled compartments: scala vestibuli, scala tympani and scala media. Scala vestibuli (containing perilymph) is separated from the scala media (containing endolymph) by the Reissner’s membrane, while scala media and scala tympani (containing perilymph) are separated by the basilar membrane. The basilar membrane contains the Organ of Corti with sensory hair cells responsible for auditory function. The hair cells are arranged in four rows along the entire length of the cochlea—three rows of outer and one row of inner hair cells. The tectorial membrane responsible for their direct (outer hair cells) and indirect (inner hair cells) activation covers the hair cells; (**C**) Schematic of a mammalian cochlear hair cell shortly after birth. Hair cells are polarized epithelial mechanosensory cells with a mechanically sensitive organelle at the apical surface, known as stereovilli (SV). Stereovilli are non-motile hair bundles consisting of dozens of specialized F-actin-filled microvilli graduated in length to form a staircase-like structure. Within their V-shaped orientation stereovilli are connected by extracellular linkages called tip links. The longest stereovilli is closest to a single genuine microtubule-based cilium, the kinocilium with a (9 × 2 + 2) microtubule configuration (which begins to regress at around P8 in the mouse). Hair cells are surrounded by non-sensory supporting cells with microvilli on their apical surface; (**D**–**H**) Transmission electron micrographs of a mouse cochlea; (**D**) Cross-section of a kinocilium (KC) showing the (9 × 2 + 2) structure; (**E**) Longitudinal section of stereovilli and the kinocilium on the apical surface of an inner hair cell; (**F**) Immature synaptic ribbon (SR) of an inner hair cell with the typical electron-dense sphere surrounded by synaptic vesicles; (**G**) V-shaped orientation of stereovilli and the kinocilium on a vestibular hair cell; (**H**) Cross-section of a synaptic ribbon in a vestibular hair cell. CS: centrosome. CP: cuticular plate. Scale bars: 50 µm (**A**,**B**), 100 nm (**D**,**H**), 200 nm (**E**,**F**), 500 nm (**G**).

The mammalian inner ear is comprised of two distinct regions: The cochlea which regulates auditory function (Figure 2A,B) and the vestibular system which perceives motion and balance. Cochlea and vestibular systems process sound and positional signals, respectively, with remarkable resolution and sensitivity. This ability depends largely on a sophisticated mechanotransduction apparatus on the surface of polarized epithelial mechanosensory hair cells and non-sensory supporting cells (Figure 2C).

At the apical surface, each hair cell contains a mechanically sensitive organelle, the hair bundle, which consists of dozens of specialized F-actin-filled microvilli, known as stereovilli or stereocilia (Figure 2C). We prefer the term stereovilli because of their non-motile character as an apical modification of the cell. Moreover, they contain actin filaments which distinguish them from microtubule-containing true cilia. Each bundle is made up of rows decreasing in height—to form a staircase-like structure—and is arranged in a V-shaped conformation with the stereovilli connected by extracellular linkages [32] (Figure 2C,E,G). The longest stereovilli is closest to a single true microtubule-based cilium, the kinocilium (9 × 2 + 2 structure) [33] (Figure 2C–E,G). The kinocilium is thought to have its main function in hair cell differentiation. During development of the hair cell bundle (mouse embryonic day 15 to postnatal day 14), the microtubule-based kinocilium emerges from the basal body at the center of the apical surface and performs a directed migration toward its final position at the lateral edge, where the stereovilli organize around it in a V-shaped bundle [34,35,36]. Disruption of kinocilium formation in cochlea-specific conditional knockout mice of the *Ift88* gene lead to abnormal morphologies, mislocalized basal bodies, and misoriented ciliary bundles. Since the direction of kinocilium migration predicts the orientation of the mature stereovilli bundle, the kinocilium is thought to be a relay station for positional information processing in the hair cells [37]. The mammalian kinocilium of cochlear hair cells does not play a role in sound transduction in the mature organ of Corti and degenerates after birth. In rat and mouse cochlear hair cells the kinocilium begins to regress at around P8, once the stereovilli bundle has formed and hearing starts [38]. However, the centrioles (previously acting as the ciliary basal body and its centriole) are retained in mature cochlear hair cells after the kinocilium has been completely reabsorbed [39]. In the non-mammalian cochlea, the kinocilium exists throughout the whole life of the animal [35]. Both kinocilia and stereovilli of mouse vestibular hair cells are considerably longer than those in the cochlea and vary in length between the different vestibular sensory organs [40]. This variation in length presumably contributes to the transduction sensitivity of these cells in the respective organs.

In summary, the kinocilium of inner ear hair cells is probably not involved in auditory perception. Nevertheless, the cochlear kinocilium is critical for the emergence of hair bundle polarity and therefore crucial for the hearing process [22]. The importance of proteins associated with cilia in audition is illustrated by Usher syndrome patients displaying deafness [30,31] and Bardet-Biedl syndrome patients displaying auditory deficiencies [28] (Table 1).

Besides their function in hearing, mammalian cilia also have an essential role in olfaction. In contrast to the kinocilium of cochlear hair cells, cilia in the olfactory system perform a direct sensory task.

## 3. The Structure and Role of Cilia in Olfaction

**Figure 3:**
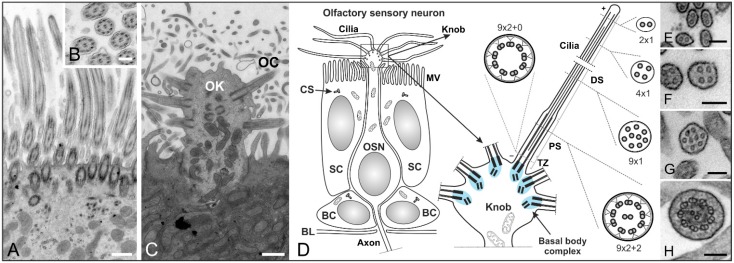
Structure of cilia in the mouse respiratory and olfactory epithelium. (**A**–**C**) Transmission electron micrographs of an adult mouse olfactory epithelium; (**A**) The respiratory epithelium contains multiple motile cilia on one cell; (**B**) Higher magnification of a cross section showing the (9 × 2 + 2) microtubule configuration of olfactory cilia; (**C**) Longitudinal section of an olfactory knob (OK) with extending olfactory cilia (OC); (**D**) Schematics of an olfactory sensory neuron (OSN) and its olfactory knob. OSNs are the receptor elements of the olfactory system. They are surrounded by supporting cells (SC) with a microvilli (MV) border on their apical surface and continually replaced by basal cells (BC) throughout life. OSNs are bipolar neurons with dendrites ending in an olfactory knob which has specialized sensory cilia responsible for olfaction. The mammalian olfactory cilium comprises the transition zone (TZ), the proximal segment (PS), and the distal segment (DS). The TZ (9 × 2 + 0 structure) is located at the base of the olfactory cilium between the basal body and the origin of the axoneme’s central pair of microtubules. The PS projects from the basal body in a (9 × 2 + 2) configuration. The DS represents the end of the cilium and contains characteristic arrays of singlet microtubules (from 9 × 1 to 2 × 1); (**E**–**H**) Higher magnification electron micrographs showing the different microtubule configurations of the DS (**E**–**G**) and PS (H) of an olfactory cilium. CS: centrosomes. BL: basal lamina. Scale bars: 500 nm (**A**,**C**), 200 nm (**B**), 100 nm (**E**–**H**).

Olfactory sensory neurons (OSN) are the receptor elements of the olfactory system. OSNs are bipolar neurons whose dendrites end in an olfactory knob, where their cilia are localized (Figure 3). OSNs are surrounded by supporting cells, which have a microvilli border on their apical surface (Figure 3C,D). In all vertebrates, olfactory receptor cells display cycles of birth, maturation and death. This turnover is remarkable given that neurons are not generally considered to undergo neurogenesis in adults. Stem cells, here called basal cells (BC), of the olfactory epithelium continually replace OSNs and non-neuronal support cells throughout life [41,42]. Cilia of the olfactory system are optimized for unique sensory function and the detection of external stimuli. Although OSNs possess 10 to 30 cilia with a (9 × 2 + 2) microtubule configuration—normally found in motile cilia—they lack the dynein arms necessary for movement and are thus rendered immotile [43,44].

**Figure 4:**
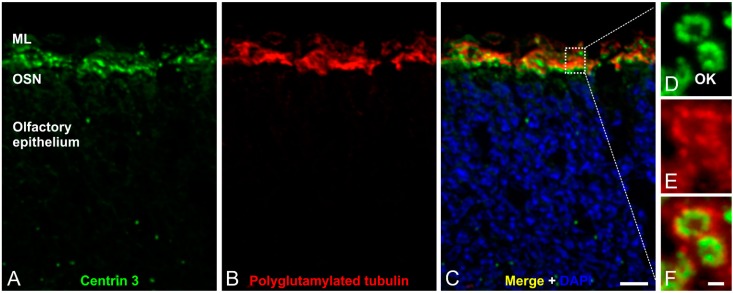
Immunofluorescence analysis of olfactory cilia in the mouse olfactory epithelium. (**A**–**C**) Confocal laser scanning micrographs of a cryostat section through adult mouse olfactory epithelium double labeled with antibodies against Centrin 3 (**green**) and polyglutamylated tubulin (**red**); (**A**,**B**) Centrin 3 and polyglutamylated tubulin are localized in the ciliary region of the olfactory sensory neurons (OSNs); (**C**) Merge combined with a DAPI nuclear staining (**blue**); (**D**–**F**) Higher magnification views of the ciliary region; (**D**) Centrin 3 is preferentially localized at the basal body, the centriole and the transition zone of cilia in the olfactory knobs (OK) of receptor neurons; (**E**) Polyglutamylated tubulin labels the axoneme of olfactory cilia; (**F**) The merge shows a partial colocalization of Centrin 3 and polyglutamylated tubulin at the transition zone of the olfactory receptor neurons. ML: mucus layer. Scale bars: 10 µm (**C** for **A**–**C**), 1 µm (**F** for **D**–**F**).

In contrast, the multiple cilia in the respiratory epithelium with a (9 × 2 + 2) microtubule backbone are motile and play a role in mucociliary clearance [3] (Figure 3A,B). The mammalian olfactory cilium is approximately 50–60 μm long—recent studies have shown the length of murine cilia to vary between 2.5 and 110 µm [45]—and can be divided into the transition zone (TZ), the proximal (PS) and the distal segment (DS) (Figure 3D–H). The TZ (“ciliary necklace”) lies at the base of the olfactory cilium, where the lipid membrane sheath contacts the dendritic knob. This domain is located between the basal body and the origin of the central pair of microtubules (9 × 2 + 0 structure, Figure 3D and Figure 4D–F) of the axoneme [1]. Interestingly, ciliary transport proteins have been found to be localized at transitional fibers (which anchor the basal body to the membrane and are located under the transition zone), indicating that the TZ might serve as a cargo-docking site connecting the ciliary shaft to the protein complex at the base of the cilium [46,47]. The PS starts 2–3 µm away from the basal body in a (9 × 2 + 2) configuration and has a diameter of around 300 nm [48,49]. The thinner distal segment spans the upper part of the cilium, with the microtubule configuration going down from 9 × 1 to 4 × 1, usually ending with a pair of singlet microtubules (Figure 3D–H). The DSes of olfactory cilia are oriented parallel to the epithelial surface. As a result, cilia from different OSNs substantially overlap which results in an enlargement of the epithelial surface and lead in turn to the detection of several odorants [48].

Olfactory cilia—like prototypic cilia—originate from a microtubule-based cell organelle, the basal body. Cells that develop a single copy cilium normally contain only one centrosome—the precursor of the basal body complex—before ciliogenesis. In contrast, OSNs multiply the centrosome in the cell body before migration to the dendritic knob. There, the modified mother centrioles move to the plasma membrane, become basal bodies and elongation of multiple cilia begins [50,51].

Olfactory sensory cilia are responsible for perception of smell. Once odorants contact the olfactory epithelium, olfaction signaling is initiated. This process starts in the long distal segment with the odorant acting as a ligand for odorant G-protein-coupled receptors (GPCRs) on the sensory neuron’s cilia.

It is generally believed that from *Drosophila* to mice to humans, each olfactory neuron expresses only one type of odorant GPCR [52,53]. In comparison, *Drosophila* has about 80, mice over 1000 and humans about 400 functional GPCR genes [52,53,54,55,56]. In mammals, odorants or mixtures of odorants bind a specific pattern of odorant GPCRs. As a result, the receptors activate adenylyl cyclase type III (ACIII) through a stimulatory G-protein (G_olf_). ACIII then generates an increase in cAMP, causing the opening of cyclic nucleotide gated (CNG) ion channels. This results in depolarization of the neuron, which is amplified via a Ca^2+^-activated chloride channel and transmitted to the brain, causing the sensation of smell [57,58,59,60]. This entire olfaction-signaling cascade takes place within the olfactory sensory cilia, with the signaling proteins, such as G_olf_, ACIII, and CNG channel, localized in the distal segment [61]. Cilia-dependent GPCR signaling is a widespread mechanism and has been widely studied over the last decade; for example, the role of vertebrate hedgehog (Hh) signaling in development and disease [62,63].

The importance of cilia in olfaction is illustrated by mouse mutants for ciliary proteins and Bardet-Biedl syndrome patients both displaying anosmia [24,28] (Table 1). In addition to their tasks in development, mechanoreception, and olfaction, another specialized group of modified cilia performs important functions in photoreception.

## 4. The Connecting Cilium as an Environmental Sensor in Light Detection

**Figure 5:**
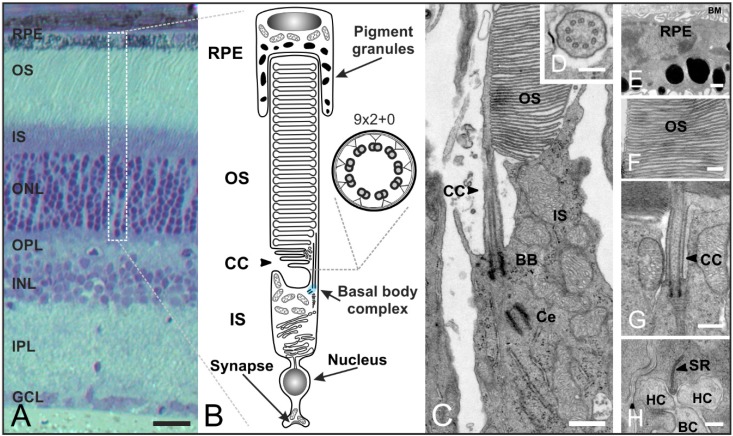
Organization of rod photoreceptors in the mammalian retina. (**A**) Vertical section of a vertebrate retina stained with toluidine blue. The retina contains five classes of neurons (photoreceptors, bipolar cells, horizontal cells, amacrine cells, ganglion cells), and is divided into three nuclear layers (outer nuclear layer (ONL), inner nuclear layer (INL), ganglion cell layer (GCL)) and two synaptic layers (outer plexiform layer (OPL), inner plexiform layer (IPL)); (**B**) Schematic of a vertebrate rod photoreceptor consisting of the long, light-sensitive outer segment (OS) enclosed by the retinal pigment epithelium (RPE) which is connected via a small intracellular bridge, the connecting cilium (CC), to the metabolically active inner segment (IS) including the basal body complex (BBC; shaded in blue) and the apical region. The OS represents a modified primary cilium. The CC corresponds to the transition zone of a prototypic cilium with a (9 × 2 + 0) microtubule array; (**C**–**H**) Transmission electron micrograph of a photoreceptor-connecting cilium; (**C**) The CC connects the OS and IS. The BBC consists of the basal body (BB) and its centriole (Ce); (**D**) Cross-section of the CC showing the characteristic (9 × 2 + 0) microtubule array; (**E**) RPE with pigment granules and the Bruch’s membrane (BM); (**F**) OS composed of stacks of tightly packed membrane discs; (**G**) Higher magnification view of the ciliary apparatus; (**H**) Synaptic ribbon (SR) with invaginations of postsynaptic horizontal cells (HC) and bipolar cells (BC). Scale bars: 100 µm (**A**), 200 nm (**D**,**F**,**H**), 500 nm (**E**,**G**).

The vertebrate retina contains five classes of neurons: photoreceptors, bipolar cells, horizontal cells, amacrine cells, and ganglion cells (Figure 5A). Out of these, photoreceptors (which are highly polarized neurons) are optimized for the detection of light. A distinction is made between rod and cone photoreceptors, which adopt different functions in vision—rod photoreceptors mediate vision in dim light, cone photoreceptors in bright light. Moreover, they can easily be distinguished by their distinctive outer segment architecture [64]. The longer outer segment (OS) of rod photoreceptors is composed of stacks of tightly packed membrane discs physically separated from the plasma membrane (Figure 5B,C,F). The outer segment of cone photoreceptors consists of folds of the plasma membrane, which are in direct contact with the extracellular milieu. Photoreceptors are divided into morphologically and functionally distinct compartments. The light-absorbing molecules are concentrated in the outer segment at the apical end of the cell and separated from the synaptic terminal (ST) at the basal end by the nucleus and inner segment (IS). The inner segment contains the typical energy-producing and protein-synthesizing components of a eukaryotic cell. The light-sensitive outer segment is linked to the inner segment by a small intracellular bridge, the connecting cilium (CC). The connecting cilium originates from the basal body complex in the inner segment and extends into the outer segment which represents the upper part of an evolutionarily modified primary cilium [65,66,67,68] (Figure 5B,C,G). In comparison to cilia in the olfactory system, photoreceptor cilia are unique in two major ways: (1) the ciliary outer segment membrane contains coin stack-shaped discs to increase the efficiency of signal detection; and (2) the outer segment discs are periodically shed distally and proximally replaced by new discs and membrane components [69].

**Figure 6:**
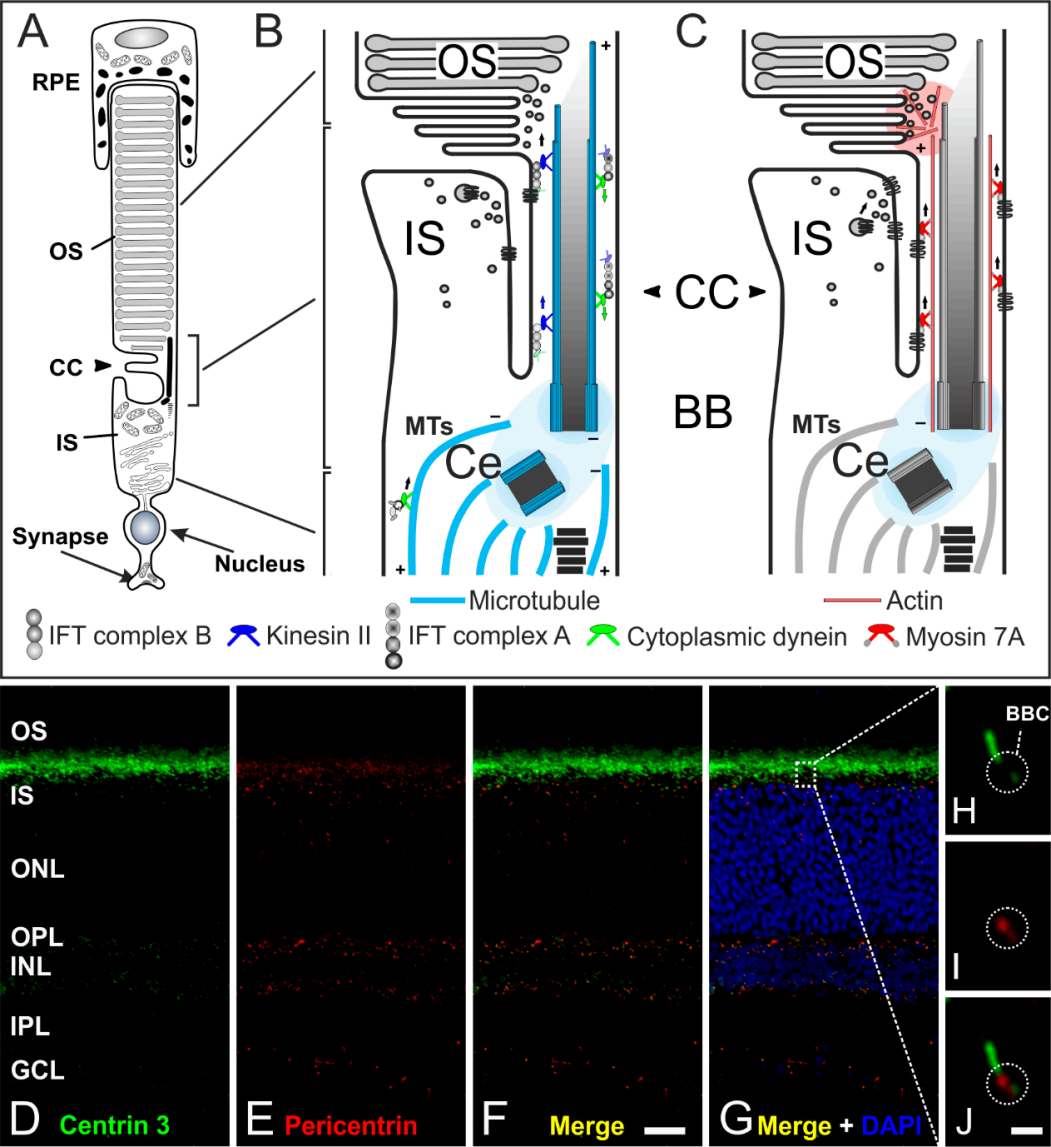
The connecting cilium of photoreceptors and its transport machinery. (**A**) Schematic of a vertebrate rod photoreceptor consisting of the light-sensitive outer segment (OS) enclosed by the retinal pigment epithelium (RPE) and linked via the connecting cilium (CC) to the metabolically active inner segment (IS); (**B**,**C**) Schematics of the two ciliary transport systems in photoreceptors; (**B**) Cargo transport to the CC is mediated along microtubules (MT) by cytoplasmic dynein 1 motor proteins (minus-end-directed). Delivery from the IS to the OS is mediated by IFT B molecules (binding the cargo) and kinesin II motor proteins (plus-end-directed). Delivery back to the IS is mediated by IFT A molecules and cytoplasmic dynein 2 motor proteins (minus-end-directed); (**C**) In addition, a myosin 7a-driven transport along actin filaments is used for trafficking proteins to the OS; (**D**–**G**) Confocal laser scanning micrographs of a vertical cryostat section through an adult mouse retina double labeled for Centrin 3 (**D**, **green**) and Pericentrin (**E**, **red**) as markers for the cilia and the basal body complex (BBC), respectively; (**F**,**G**) As seen in the merge of the stainings with additional labeling of the cell nuclei with DAPI, centrin 3 and pericentrin partially colocalize at the ciliary region of the photoreceptors and at the centrosomes of other retinal cells; (**H**–**J**) Higher power views showing the partial colocalization of centrin 3 and pericentrin at the BBC of photoreceptor-connecting cilia. Scale bars: 20 µm (**F**), 1 µm (**J**).

The following description of transport processes will focus on rod photoreceptors as the mechanisms are understood best in these cells. The signal transduction cascade as well as the high membrane turnover in the OS require an efficient transport of disc components from the nuclear region and the IS to the OS [70] (Figure 6A–C). For example, the visual pigment rhodopsin is the most abundant protein in the OS (85% of total OS protein) and an enormous number of molecules (10^4^–10^7^) are synthesized by rod photoreceptors every day [65,67]. Rhodopsin is the light receptor that activates the phototransduction cascade, which results in membrane hyperpolarization and inhibition of synaptic transmission [71,72,73,74,75,76,77]. But how do photoreceptors realize this permanent transport of proteins? All the proteins synthesized in the IS but destined for the OS have to be delivered to the basal body complex for distal trafficking through the narrow non-motile-connecting cilium (Figure 6A–C). The connecting cilium is the structural equivalent of the transition zone of motile cilia and flagella (prototypic cilium) with the characteristic (9 × 2 + 0) microtubule array [65,66,78] (Figure 5B–D). The connecting cilium expands to the outer segment which represents the upper part of the evolutionarily modified primary cilium. Development of the photoreceptor outer segment and connecting cilium is mediated by IFT transport processes (well described for rod photoreceptors in [79]). In mature rod photoreceptors, we can distinguish between two types of molecular transport systems: (1) a myosin 7a motor transport system along actin filaments [66,80,81]; and (2) the microtubule-based intraflagellar transport system composed of IFT molecules (binding the cargo), kinesin II motor proteins (plus-end-directed) and cytoplasmic dynein 2 motor proteins (minus-end-directed) for transport [68,78,82] (Figure 6A–C). How the two distinct motors and the cytoskeletal systems are coordinated remains unknown. All components of the phototransduction cascade—such as rhodopsin, rhodopsin kinase, cyclic nucleotide gated channels, and phosphodiesterases—have to be transported to the OS. For instance, rhodopsin is synthesized and modified in the endoplasmic reticulum (ER). Then the visual pigment is packed into so-called rhodopsin transport carriers (RTCs) in the Golgi [68,83]. These RTCs are uni-directionally transported by cytoplasmic dynein 1 along microtubules to the base of the connecting cilium [84] (Figure 6B). There, they fuse with the plasma membrane surrounding the connecting cilium through which rhodopsin is then delivered to the rod outer segment [83]. The proteins arrestin and transducin undergo light-dependent translocation processes between the inner and outer segment, and must therefore be transported in both directions [85,86,87]. Many studies have shown that cone and rod photoreceptors possess distinct transport machineries and pathways for trafficking proteins to the outer segment (and back) [69,88,89]. In summary, the development and maintenance of photoreceptor cell function requires effective and well-regulated transport processes along the connecting cilium. Thus, the connecting cilium is the passage between the inner and outer segment with roads in both directions and a traffic control system for different cargos [90] (Figure 6A–C).

## 5. Concluding Remarks

Recently, cilia have received increasing attention as they are involved in multiple cellular functions and adopt crucial roles in vertebrate development. Ciliary malfunctions can result in human diseases called ciliopathies [3,10] (Table 1). The complexity of this organelle and the wide array of sensory and signaling activities that it plays a role in have raised many questions [59]. In this review, we focused on structure, development, and specialized sensory functions of cilia in mammalian sensory systems. In the mammalian auditory system, hair cells possess one single non-motile (9 × 2 + 2) kinocilium which is located at the end of a V-shaped bundle of actin-rich stereovilli. This kinocilium seems to be critical for the development of hair bundle polarity and is therefore indirectly involved in the hearing process. In the mammalian olfactory epithelium, multiple long non-motile cilia with a (9 × 2 + 2) structure are present in one cell, which contains the entire signal-processing machinery necessary for olfaction. In contrast to cilia in hair cells and olfactory sensory neurons, photoreceptors possess a connecting cilium, which corresponds to the transition zone of a prototypic cilium with a (9 × 2 + 0) microtubule array. It connects the metabolically active inner segment with the outer segment which contains the signal transduction machinery and evolutionarily represents the axonemal part of a modified primary cilium.

Mutations in ciliary proteins of the inner ear, the olfactory epithelium, and the retina result in a wide array of human diseases. These ciliopathies are characterized by sensory impairments like deafness, anosmia, and retinal degeneration (Table 1). Mouse models have taught us a great deal about the pathogenesis of ciliopathy phenotypes and are withal the most important tool for studying human health and disease (a detailed overview of mouse mutants of ciliopathy genes is shown in [91]).

**Table 1:** Ciliopathies affecting sensory systems. Table includes some of the most notable human syndromic disorders affecting sensory organs like the inner ear, the olfactory epithelium, and the retina, in addition to the genes that have been linked to the diseases. The Mouse Genome Informatics (MGI) database (http://www.informatics.jax.org) provides information about all mouse models known to date to investigate human health and disease.

Ciliopathy or Related Disease	Associated Genes	Affected Tissues	Symptoms in Mouse and Human	Representative Mouse Mutants	Key References
Alström syndrome	*ALMS1*	Eyes, inner ear, adipose tissue	Blindness, hearing loss, obesity	*Alms1*^−/−^	[92,93]
Bardet-Biedl syndrome	*BBS1-BBS16*	Eyes, olfactory system, kidneys, bone, central nervous system, adipose tissue	Blindness, anosmia, renal dysfunction, polydactyly, obesity, mental retardation	*Bbs1*^−/−^	[24,94,95]
				*Bbs3*^−/−^	
				*Bbs4*^−/−^	
Joubert syndrome	*JBTS1-JBTS15*	Brain, eyes, olfactory system, muscle, kidney, liver, bone	Mental retardation, blindness, anosmia, renal and liver dysfunction, uncoordinated movements	*Cep290^rd16^*	[96,97]
				*Arl13b^hnn^*	
Mainzer-Saldino syndrome	*IFT140*	Eyes, bone, kidney	Blindness, phalangeal cone-shaped epiphyses, abnormality of the proximal femur, renal dysfunction	*Ift140^cauli/cauli^*	[98,99,100]
MOPD II and Seckel syndrome	*PCNT* *POC1A*	Olfactory system, internal organs	Anosmia, developmental defects, dwarfism	*Pcnt^ocd^*	[101,102,103]
				*Pcnt*^−/−^	
				*Poc1a^em1J^*	
Senior-Loken syndrome	*NPHP1* *NPHP4* *NPHP6*	Eyes, internal organs, reproductive system	Blindness, male infertility heterotaxy	*Cep290^rd16^*	[96]
Usher syndrome	*USH1A-G* *USH2A-C* *USH3A-B*	Inner ear, eyes, reproductive system	Hearing loss, blindness, infertility, movement anomalies	*Ush1c*^−/−^	[104,105,106]
				*Waltzer^2J^*	

In all cilia, transport processes adopt important roles in ciliary development and function. Best known is the protein transport mediated by IFT and its molecular motors. In addition, other transport systems might also be important for ciliary function, such as an actin- and myosin-dependent transport which has thus far only been reported in photoreceptors.

In this review, it was our intention to show that cilia adopt very important roles in various sensory tissues. Often, only one mutation in a ciliary protein leads to deficient formation and dysfunction of cilia, resulting in sensory impairments. Our group is currently focusing on the characterization of proteins located at the basal body complex of sensory cilia, e.g., Pericentrin (Figure 6D–J) [18,107,108,109]. The basal body complex, which is enclosed by the pericentriolar material (PCM), seems to be the organizing center of protein transport into and out of the cilium and might be the key to connect ciliary function to the phenotypes of numerous ciliopathies. In particular, the interactions between the different protein networks as well as their side branches are not fully understood. Future analysis of the molecular interactome at photoreceptor cilia and cilia in general will certainly provide new insights into the function and dysfunction of cilia in health and disease.
